# Biomarcadores de função endotelial em doenças cardiovasculares: hipertensão

**DOI:** 10.1590/1677-5449.000316

**Published:** 2016

**Authors:** Josynaria Araújo Neves, Josyanne Araújo Neves, Rita de Cássia Meneses Oliveira

**Affiliations:** 1 Universidade Federal do Piauí – UFPI, Núcleo de Pesquisa em Plantas Medicinais – NPPM, Teresina, PI, Brasil.

**Keywords:** biomarcadores, hipertensão, função endotelial, ADMA

## Abstract

A incidência de hipertensão arterial sistêmica está aumentando mundialmente. Sua prevenção baseia-se na identificação dos hipertensos. Atualmente, biomarcadores são utilizados com fins de diagnosticar, estratificar e prognosticar doenças. Neste estudo, objetivou-se revisar artigos dos últimos cinco anos relacionados a biomarcadores nas doenças cardiovasculares. Pesquisaram-se dados de PubMed, SciELO, Science Direct e MEDLINE, mediante as palavras-chave: hipertensão arterial, biomarcadores cardiovasculares, óxido nítrico, função endotelial e dimetilarginina assimétrica. Os estudos levantados mostram que as doenças cardiovasculares possuem uma etiologia complexa. Neste artigo, evidenciaram-se interações entre o óxido nítrico e a dimetilarginina assimétrica na regulação, no metabolismo e na determinação dos níveis intracelulares, e reviram-se outros biomarcadores relacionados à hipertensão. Alguns estudos indicam os biomarcadores como uma ferramenta útil na predição de eventos cardíacos, e outros reportam que eles contribuem pouco para a avaliação. A seleção e combinação desses pode ser uma alternativa para validar o uso dos biomarcadores devido à pouca especificidade existente para diagnosticar a hipertensão.

## INTRODUÇÃO

A hipertensão arterial sistêmica (HAS) constitui um problema de saúde pública grave em virtude de sua dimensão, risco e dificuldades no controle, e está relacionada a uma elevada taxa de mortalidade por promover o desenvolvimento de doenças cardiovasculares (DCVs)^1^. As DCVs atingem mais de 83,6 milhões de norte-americanos e, no Brasil, o Ministério da Saúde verificou a ocorrência de 326 mil casos de morte por essas doenças, o que corresponde a cerca de 1.000 mortes por dia em 2010^2^.

De acordo com Georgiopoulou et al.^3^, a incidência de HAS aumentou globalmente. Para Durande e Gutterman^4^, as células endoteliais incorporam uma gama de funções homeostáticas. Tem sido sugerido que a disfunção do endotélio vascular associado à HAS está relacionada com uma inflamação local e sistêmica^5^. Sabe-se que a inflamação é uma resposta fisiológica protetora a estímulos nocivos e/ou patogênicos, e que a disfunção endotelial é um estado pró-inflamatório, com alteração nas funções do endotélio, e está associada à HAS, uma doença multifacetada.

Entre os fatores patológicos, podem-se citar os radicais livres, que promovem lesão tecidual e disfunção endotelial por perturbarem o equilíbrio do óxido nítrico (NO), causando elevado estresse oxidativo, elevados níveis de citocinas pró-inflamatórias (fator de necrose tumoral alfa - TNF-α; interleucinas - IL-6 e IL-1β) e a produção excessiva de quimiocinas inflamatórias [macrófago inflamatório proteína alfa-1 (MIP-1α) e quimioatrator proteína-1 (MCP-1)]^6^^,^ 7.

A patologia HAS constitui um fator de risco relacionado à alta morbidade e mortalidade por contribuir para o agravamento de outras DCVs e doenças renais^8^. Assim, a HAS está nitidamente associada ao desenvolvimento de lesões vasculares e ao aparecimento de disfunções em órgãos-alvo como encéfalo, coração, vasos sanguíneos e rins^9^, levando a complicações como: acidente vascular encefálico, infarto agudo do miocárdio, insuficiência cardíaca, doença arterial periférica, doença arterial coronariana e doença renal crônica.

Os biomarcadores são comumente empregados na medicina clínica de DCVs tanto para diagnosticar e estratificar os riscos como também para prognosticar tais patologias^10^. Como exemplo do uso de biomarcadores, pode-se citar a dosagem de osteoprotegerina em casos de insuficiência cardíaca, que pode estar relacionada à HAS em decorrência de ser a causa da hipertrofia do miocárdio^11^.

Objetivou-se por meio do presente estudo revisar na literatura artigos científicos relacionados ao tema HAS e biomarcadores. Durante os últimos 10 anos, a dimetilarginina assimétrica (ADMA) vem se destacando como um biomarcador cardiovascular promissor. Nesse contexto, buscou-se relatos de ADMA no plasma como um novo biomarcador de risco cardiovascular putativo, como na hipertensão, assim como de contribuições da incorporação de outros modelos de biomarcadores, como células progenitoras de endotélio, troponina T, vitamina D e ácido úrico.

Assim, foi realizada uma revisão da literatura atual utilizando referências bibliográficas buscadas nas bases de dados PubMed, SciELO, Science Direct, e MEDLINE, publicadas durante o período de 2010 a 2015, por meio da utilização e combinação das seguintes palavras-chave: hipertensão arterial, biomarcadores cardiovasculares, óxido nítrico, função endotelial e dimetilarginina assimétrica. Foram pesquisados, também, os mesmos termos no idioma inglês.

## PRESSÃO ARTERIAL E HIPERTENSÃO

A pressão arterial (PA) pode ser definida como a força exercida pelo sangue por unidade de área da parede vascular. Essa pressão gerada pelo coração é a força (energia potencial) que permite a ocorrência do fluxo sanguíneo e a perfusão sanguínea nos tecidos. Por intermédio do fluxo circulatório, são fornecidos aos órgãos e tecidos quantidades de oxigênio conforme a necessidade e retiram-se os metabólitos resultantes da atividade celular. Dessa forma, a manutenção de uma pressão adequada é fundamental para que ocorra o bom funcionamento do sistema circulatório.

A fisiopatologia da HAS implica em entender os mecanismos de controle da PA, cuja regulação depende de ações integradas entre os sistemas cardiovascular, renal, neural e endócrino, e de fatores físicos como força contrátil do coração e elasticidade das grandes artérias torácicas. De acordo com Franceschini et al.^8^, a manutenção da PA dentro de uma faixa de normalidade depende de variações no débito cardíaco e na resistência periférica, com inúmeras substâncias e sistemas fisiológicos interagindo de maneira complexa para garantir uma PA em níveis adequados nas mais diversas situações.

A HAS é uma desordem circulatória^12^. Essa condição clínica multifatorial está associada a alterações metabólicas e funcionais/estruturais dos órgãos-alvos (coração, rins e vasos sanguíneos), caracterizada por níveis elevados e sustentados de PA, geralmente acima da meta (nível pressórico sistólico ≥ 140 mmHg e/ou nível pressórico diastólico ≥ 90 mmHg)^13^^,^ 14. A HAS, ou o aumento da PA, é reconhecida como um importante fator de risco para a morbidade e mortalidade cardiovascular^15^^,^ 16, mesmo com o uso concomitante de medicamentos anti-hipertensivos.

A literatura reporta alguns fatores ambientais relacionados com a progressão, evolução e complicações nas patologias cardiovasculares, e entre esses fatores incluem-se o tabagismo, a obesidade, o sedentarismo e outros. A combinação desses fatores entre indivíduos hipertensos parece variar com a idade, a inatividade física, a hiperglicemia e a dislipidemia, sendo que a obesidade aumenta a prevalência da associação de múltiplos fatores de risco^14^. Lima et al.^17^, em estudo com pacientes brasileiros submetidos a revascularização miocárdica, concluiu que a hipertensão arterial, a obesidade e o sedentarismo foram os fatores mais frequentemente encontrados, e que 79,5% dos pacientes apresentaram, no mínimo, três fatores de risco. Observou-se ainda que os fatores que promovem alterações na PA vêm ocorrendo em faixa etária cada vez mais baixa^18^.

Diante do exposto, Nobre et al.^19^ ressaltam que a gênese da hipertensão compreende aspectos genéticos, ambientais, vasculares e neurais. A hiperatividade do sistema nervoso simpático é proposta como um importante mecanismo de hipertensão e DCV^20^.

Determinantes sociais, como urbanização, renda, envelhecimento e educação, influenciam o desenvolvimento dessa patologia^12^. Portanto, para prevenção da HAS, alterações no estilo de vida são recomendadas pelas diretrizes nacionais e internacionais, que nos últimos anos vêm se fortalecendo devido ao aumento da sobrevida dos pacientes com doenças crônicas^15^.

Entre os mecanismos fisiopatológicos da HAS, podemos citar o papel do endotélio vascular.

## ENDOTÉLIO E ALTERAÇÕES FISIOPATOLÓGICAS

As células endoteliais, em condições fisiológicas, são controladas por fatores hemodinâmicos, como PA e fluxo sanguíneo, que levam a uma resposta, dependente da produção de mediadores químicos, e que produzem modificações no fluxo sanguíneo^21^. Essas células desempenham um papel fundamental na regulação do tônus vascular através da síntese e liberação de fatores de relaxamento e contração envolvidos na homeostase cardiovascular^22^.

De acordo com Dias et al.^23^, os principais fatores relaxantes derivados do endotélio são o NO, o fator hiperpolarizante derivado do endotélio (EDHF) e a prostaciclina (PGI2). Entre os fatores contráteis, os principais são a prostaglandina H2 (PGH2), a tromboxana A2, a angiotensina II (Ang II), as espécies reativas de oxigênio (ROS) e a endotelina (ET-1).

O endotélio é um órgão com múltiplas funções. A habilidade endotelial de modular o lúmen vascular pela regulação do tônus vascular (controle da dilatação e da contração local) ou pela liberação de fatores vasoativos é uma das principais respostas aos estímulos fisiológicos gerados pelo fluxo e pela pressão sanguínea^24^^-^ 27.

Na hipertensão, o complexo mecanismo de elevação da PA pela deficiência de NO envolve acentuação do tônus do sistema simpático, do sistema renina-angiotensina (RAS) e do estresse oxidativo^28^^,^ 29. Todas as células, incluindo as endoteliais, possuem complexos sistemas antioxidantes enzimáticos e não enzimáticos, que atuam sinergicamente para defender o organismo de danos causados pelos radicais livres^30^.

**Figura 1:**
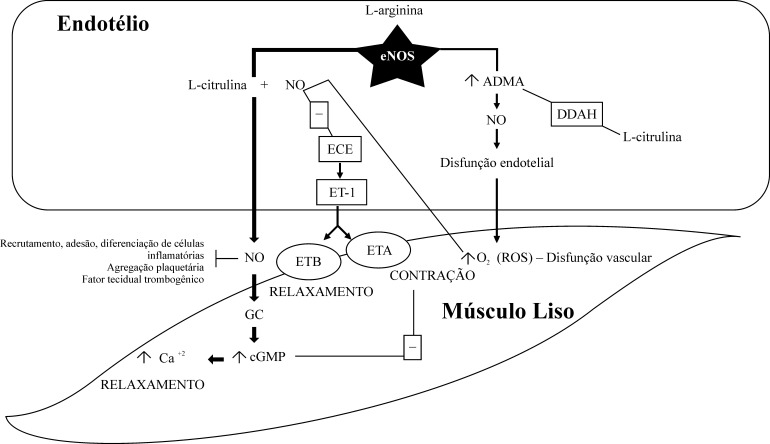
Sinalização endotelial e mecanismo de relaxamento ou contração do músculo liso. eNOS: enzima óxido nítrico sintase endotelial; NO: óxido nítrico; GC: guanilato-ciclase; cGMP: guanosina monofosfato cíclico; Ca^2+^: íons de cálcio; ECE: enzima de conversão da endotelina; ET-1: endotelina 1; ETA, ETB: receptores das endotelinas – ETA e ETB em células de músculo liso vascular; O_2_: radicais livres; ROS: espécies reativas de oxigênio; ADMA: dimetilarginina assimétrica; DDAH: dimetilarginina-dimetilamino-hidrolase.

O NO é um radical livre inorgânico, gasoso, altamente reativo e produzido a partir da oxidação do aminoácido L-arginina, que se converte em L-citrulina^23^. O NO é gerado a partir da L-arginina pela ação de NO-sintase endotelial, na presença de cofatores. Uma vez que ele é produzido, difunde-se para células musculares lisas vasculares e ativa a guanilato-ciclase (GC), o que leva à vasodilatação mediada pela liberação do segundo mensageiro guanosina monofosfato cíclico (cGMP) e à ativação da proteína quinase G-dependente, resultando na diminuição da concentração de cálcio intracelular, seguida por vasorrelaxamento. Além disso, há muitas outras funções do NO que participam da regulação de transcrição de genes, tradução de mRNA e modificação proteica de várias enzimas envolvidas na respiração mitocondrial, na mitogênese e no crescimento^31^. O sistema de sinalização NO e cGMP é um modulador bem caracterizado da função cardiovascular, em geral, e da PA, em particular^32^ (Figura 1).

Existem três isoformas da enzima responsável pela síntese de óxido nítrico (NOS). A forma neuronal (nNOS), encontrada nos neurônios e ativada por cálcio, a forma induzível (iNOS), estimulada por citocinas inflamatórias, produtos microbianos e perturbação mecânica^33^, e a enzima óxido nítrico sintase endotelial (eNOS), localizada no cromossomo 7 (7q35-36), são responsáveis pela síntese de NO na circulação sanguínea, com capacidade vasoprotetora e vasodilatadora^34^. Além de inibirem a proliferação das células musculares lisas, impedem o recrutamento, a adesão e a diferenciação de células inflamatórias, a agregação plaquetária e a produção do fator tecidual trombogênico^21^^,^ 35.

A atividade de eNOS e de nNOS é dependente do complexo cálcio/calmodulina (Ca^2+/^CaM). Desse modo, ela é controlada pela variação de concentração de cálcio intracelular, que é um importante sinalizador citoplasmático. A iNOS independe do aumento das concentrações intracelulares de Ca^2+^, segundo Forsterman e Sessa^31^, sendo expressa em processos celulares anormais induzidos e/ou estimulada por citocinas, agentes inflamatórios e perturbação mecânica, resultando em alto fluxo de NO^23^^,^ 34.

## BIOMARCADORES DE NO/ADMA

Defeitos na função endotelial e na produção de NO foram associados com aterosclerose, hipertensão, diabetes, obesidade, sedentarismo, tabagismo, idade avançada, baixa biodisponibilidade de L-arginina e presença de agentes infecciosos^36^.

Estudos com metabólitos do NO, como nitrito/nitrato (NOx), mostram que eles atuam monitorando o estado de saúde de pacientes com DCVs e podem ser utilizados no campo clínico como biomarcadores^37^. Rajendran et al.^38^ corroboram essa abordagem sobre biomarcadores, pois avaliaram a função endotelial baseados na medição dos biomarcadores endoteliais circulantes no plasma, uma vez que existem muitos candidatos atrativos para biomarcadores endoteliais. No entanto, os autores ressaltam que, em muitas patologias, essas moléculas possuem uma fraca seletividade e especificidade. Assim, se forem utilizadas individualmente, têm um baixo valor preditivo.

Considerando que a produção de NO foi associada à baixa biodisponibilidade de L-arginina^36^ e que este substrato é análogo à ADMA, estudos sugerem que a razão L-arginina/ADMA tem reflexo direto na biodisponibilidade de NO^39^. No plasma de indivíduos saudáveis, a relação L-arginina/ADMA é de aproximadamente 100:1, segundo Sharma et al.^40^. Porém, em uma situação fisiopatológica, a ADMA apresenta concentrações aumentadas quando comparada à L-arginina; dessa maneira, a ADMA pode atuar inibindo competitivamente a eNOS, uma vez que inibe a formação de NO e, consequentemente, reduz a síntese desse substrato^41^. Assim, os níveis elevados de ADMA podem inibir a síntese de NO, prejudicando a função endotelial.

Os níveis plasmáticos elevados de ADMA têm sido relatados em pacientes com fatores de risco vascular, possuindo valor preditivo para eventos cardio e cerebrovasculares, sendo que Nishiyama et al.^42^ destacaram a ADMA como um biomarcador da hipertensão arterial. Com base nesta pesquisa bibliográfica, ressalta-se o déficit de estudos sobre os efeitos dos níveis intracelulares de ADMA na hipertensão nos últimos cinco anos.

Estudos revelaram ainda que níveis elevados de ADMA reduzem a participação do NO na regulação do tônus vascular por atuarem na inibição direta da eNOS e na diminuição da biodisponibilidade desta pelo aumento da produção e liberação de ROS através da ativação do RAS, levando a uma disfunção vascular em vasos arteriais isolados *in vitro* 41.

Evidências sugerem que o desequilíbrio nos níveis de NO e ROS (diminuição e aumento, respectivamente) ativa o sistema nervoso simpático, mecanismo que parece estar envolvido nos aspectos neurogênicos da hipertensão^43^. Desse modo, a ADMA pode servir como um biomarcador, indicando a redução da biodisponibilidade do NO^41^.

## BIOSSÍNTESE DE ADMA

**Figura 2:**
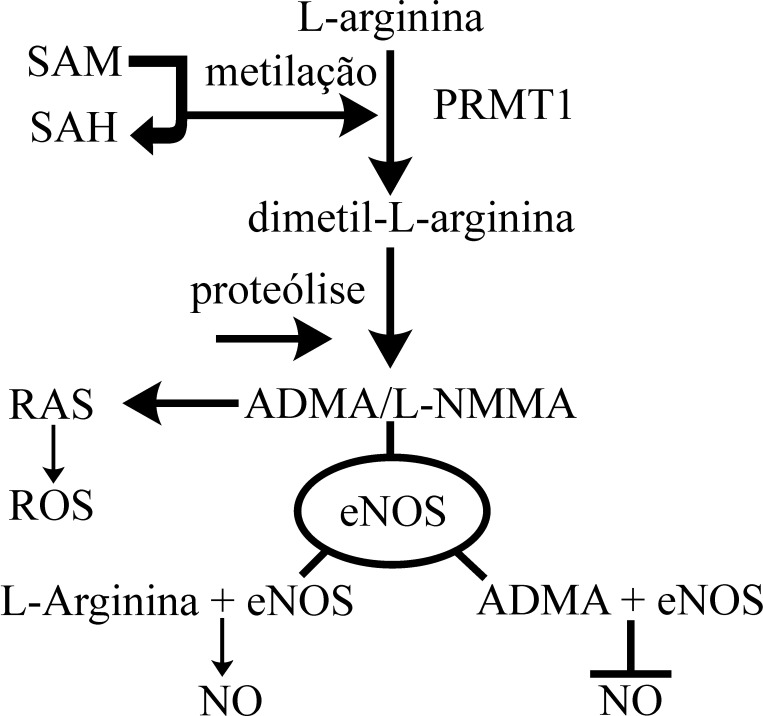
Via da ADMA e sua relação com o NO. ADMA: dimetilarginina assimétrica; SAM: sadenosilmetionina; SAH: S-adenosil; PRMT1: enzima metiltransferase tipo 1; SDMA: dimetilarginina simétrica; L-NMMA: NG-monometil-L-arginina; RAS: sistema renina-angiotensina; ROS: espécies reativas de oxigênio; eNOS: enzima óxido nítrico sintase endotelial; NO: síntese de óxido nítrico.

O substrato ADMA é formado depois da proteólise de proteínas contendo resíduos de arginina metilados. Essa metilação é facilitada pela enzima metiltransferase (PRMTs), que utiliza a proteína S-adenosilmetionina (SAM) como o grupo doador de metilo^44^. Sabe-se que existem dois tipos de PRMTs, de acordo com a atividade catalítica específica: tipo 1, que catalisa a formação de ADMA e NG-monometil-L-arginina (L-NMMA); e tipo 2, que catalisa a formação de dimetilarginina simétrico (SDMA) e L-NMMA (Figura 2).

Atualmente, destaca-se a ADMA degradada pela dimetilarginina-dimetilamino-hidrolase (DDAH)^45^, constituída de duas isoformas (DDAH-1 e DDAH-2). A degradação da ADMA pela isoforma enzimática DDAH-1 ocorre nos tecidos^46^, sendo proposto que a modulação da atividade DDAH-1 através dos agonistas do receptor farnesoid X (FXR), a exemplo 3-(2,6-diclorofenil)-4-(3'-carboxi-2-chlorostilben-4-il)-oximetil-5-isopropylisoxazole (GW4064), pode ser utilizada como um alvo terapêutico para o tratamento de insuficiência cardíaca congestiva e outras DCVs^47^.

Para Caplin et al.^48^, 80 a 90% de ADMA é metabolizada principalmente por DDAHs. Uma via alternativa ocorre por alanina-aminotransferase 2 glioxilato (AGXT2) no rim^49^. A ADMA, por acumular-se no plasma, pode ser considerada um fator patofisiológico de DCVs e também renais.

Segundo Davids et al.^50^, é preciso ter em mente que os níveis circulantes de ADMA, em alguns casos, refletem as concentrações intracelulares. Contudo, esses níveis não estão em equilíbrio^51^.

Em pacientes cardíacos, de acordo com Nemeth et al.^45^, níveis elevados de ADMA no líquido pericárdico podem indicar mecanismos fisiopatológicos importantes, como a redução da biodisponibilidade do NO, contribuindo para o desenvolvimento de hipertrofia cardíaca e remodelação. Assim, os autores propõem que a análise desse líquido pode ser uma ferramenta de diagnóstico por interferir no conteúdo e nos efeitos do líquido pericárdico, abrindo novas opções terapêuticas para modificar beneficamente a função e a estrutura cardíaca.

Sabe-se que a ADMA foi identificada como um fator de risco para a disfunção endotelial que age em várias DCVs acelerando a progressão^52^. Estudos recentes em pacientes com doença renal crônica (DRC) sugerem uma relação ou associação entre os níveis de ADMA e o fator de crescimento de fibroblastos 23 (FGF-23), além de uma relação com marcadores de lesão da célula endotelial^53^^,^ 54.

Vários estudos que avaliaram biomarcadores convencionais e inovadores para a predição de eventos cardiovasculares foram relatados. Reriani et al.^55^ reportam a necessidade de averiguação da utilidade dos biomarcadores putativos na avaliação do risco cardiovascular, quando comparados com a função endotelial, devido ao pequeno valor incremental de biomarcadores sobre os fatores de risco convencionais. Seria especular que biomarcadores em tais estudos ainda iriam desempenhar um papel mínimo na estratificação de risco cardiovascular.

## OUTROS BIOMARCADORES

Na Tabela 1, foram apresentados alguns biomarcadores amplamente conhecidos. Segundo Hirata et al.^56^, vários marcadores já foram citados na literatura sobre a disfunção endotelial vascular, como insulina, adiponectina, vasodilatadores (nitrito e nitrato) e vasoconstritores (ROS, endotelina, tromboxana A2). No presente estudo, foram encontradas pesquisas direcionadas para a função endotelial e a investigação de outros possíveis biomarcadores, apresentados a seguir.

**Tabela 1:** Alguns marcadores dosados apresentam relação com o risco cardíaco.

Categoria	Biomarcador	Efeitos
Inflamação vascular	PCR^*^ (proteína)	- Promove disfunção endotelial por suprimir diretamente a produção de NO^†^ - Ativação do RAS^‡^ - Aumento dos níveis de PCR foram associados com risco aumentado de hipertensão
	IL-6^§^, IL-1||, TNF-α^¶^, MCP-1^**^ (citocina)	- IL-6 se correlaciona positivamente com as pressões sistólica e diastólica - A concentração de IL-6 e PCR coincide com aumento do IMC^††^, de lipídeos e de outros fatores de risco relacionados com a aterosclerose
Estresse miocárdico	BNP^‡‡^ e NT-proBNP^§§^ (neuro-hormônio)	- Dilatação ventricular e sobrecarga de pressão na parede miocárdica - Aumento da taxa de filtração glomerular - Aumento da excreção de água e sódio
Estresse oxidativo e função renal	Homocisteína	- Aumento da homocisteína no plasma como um marcador de risco cardiovascular associado a idade, colesterol e hipertensão
Potencial fibrinolítico	PAI-1||||	- Inibidor de serino-protease produzido por células endoteliais - Aumento dos níveis de PAI-1 em indivíduos hipertensos como resultado da indução do cisalhamento e/ou ativação endotelial

* PCR: proteína C-reativa.

† NO: óxido nítrico.

‡ RAS: sistema renina-angiotensina.

§ IL-6: interleucina-6.

|| IL-1: interleucina-1.

¶ TNF-α: fator de necrose tumoral-α.

** MCP-1: quimioatrator proteína-1.

†† IMC: índice de massa corporal.

‡‡ BNP: peptídeo natriurético tipo B.

§§ NT-proBNP: pró-BNP.

|||| PAI-1: fator ativador de plasminogênio 1.

### Células Progenitoras de Endotélio (CPEs)

As CPEs são células mononucleares que expressam uma combinação de marcadores endoteliais (VEGFR2) e progenitores (CD34/CD133), com alta capacidade proliferativa, podendo ser isoladas do sangue periférico, medula óssea e sangue do cordão umbilical. Não há um único marcador específico para a identificação dessas células, sendo atualmente o mais aceito a coexpressão de marcadores CD133, CD34 e VEGFR2. Embora esses marcadores não sejam exclusivos de CPE, a sua combinação caracteriza uma célula progenitora específica em relação a um estágio de maturação específico^57^^,^ 58.

Descobriu-se que as CPEs auxiliam no processo do reparo endotelial e na formação de novos vasos, o que as condiciona a serem utilizadas como marcador de saúde cardiovascular.

### Troponina T (hs-cTnT)

McEvoy et al.^59^ avaliaram a sensibilidade de troponina T (hs-cTnT), um marcador de lesão miocárdica subclínica que pode identificar indivíduos com risco de hipertensão ou hipertrofia ventricular esquerda. Eles concluíram que a hs-cTnT está associada com a incidência de hipertensão e o risco de hipertrofia ventricular esquerda em uma população ambulatorial e sem história de DCV. Contudo, os autores ressaltam a necessidade de mais pesquisas para determinar se a hs-cTnT pode identificar e beneficiar a monitorização ambulatorial da PA ou hipertensão.

Autores com Bugnicourt et al.^60^ e Tu et al.^61^ demonstraram que as concentrações de troponina podem prever um novo início de fibrilação atrial, uma vez que está independentemente associada com pior resultado do curso por causa de eventos adversos cardíacos graves. O mecanismo exato pelo qual a fibrilação atrial leva à elevação de hs-TN não é totalmente compreendido e mais estudos são necessários. Contudo, biomarcadores como hs-TNI ou hs-TNT são de fácil manuseio e de menor valor financeiro.

O uso de hs-TN em pacientes que estão em risco muito elevado poderia identificar e, consequentemente, receber um tratamento especial com uma maior taxa de controle e uma definição mais rigorosa dos limites de possíveis fatores de risco, como PA, hipercolesterol e diabetes melito^62^. Sabe-se que os valores de hs-TNI são muito sensíveis na identificação de danos no tecido do coração.

### Vitamina D

Os efeitos da vitamina D na homeostase do cálcio e no metabolismo ósseo foram bem ressaltados na literatura. Contudo, estudos mais recentes reportam sua associação a diversos problemas de saúde, como os efeitos sobre a saúde cardiovascular, uma vez que a vitamina D pode interagir com vários mecanismos.

De acordo com os autores Brondum-Jacobsen et al.^63^, Grandi et al.^64^, Sokol et al.^65^ e Wang et al.^66^, os níveis de vitamina D podem atuar como biomarcadores em pacientes com DCV.

A principal vitamina D circulante apresenta-se na forma de 25-hvitamina D. Essa forma circulante liga-se diretamente ao receptor da vitamina D (VDR) para exercer os seus efeitos ou pode ser convertida, no rim, por 1 α-hidroxilase a 1,25-di-hidroxivitamina D, também conhecida como hormônio calcitriol^67^^,^ 68. Deve-se lembrar que o calcitriol atua através da ligação com a VDR, que está presente no endotélio vascular, o músculo liso vascular e o miocárdio, o que salienta a possibilidade de um efeito biológico direto da vitamina D no sistema cardiovascular. A maioria dos estudos reportam que baixos níveis de 25-hidroxivitamina D estão associados a fatores de risco cardiovascular, como hipertensão, diabetes e inflamação.

### Ácido Úrico (AU)

Atualmente, o AU é uma das condições mais estudadas em relação à HAS. Yanik e Feig^69^ demonstraram uma associação entre hiperuricemia e hipertensão arterial, corroborando o uso do nível sérico como um biomarcador para o diagnóstico.

A formação de AU ocorre a partir da quebra de adenosina e guanina via hipoxantina, que se converte em xantina e AU por meio da xantina oxidase^70^. As reações catalisadas por xantina oxidase geram ROS como subprodutos, como o ânion superóxido, que reage com prótons e NO para originar novas ROS. Estas, por sua vez, promovem lesão do endotélio cardiovascular e da microvasculatura^71^.

Mamas et al.^72^ ressaltam que a maioria dos biomarcadores não são metabólitos, como as troponinas, que são proteínas. A análise dos metabólitos dos fluidos corporais tornou-se uma parte importante do diagnóstico, prognóstico e avaliação das intervenções terapêuticas na aplicação clínica, uma vez que o metaboloma é o produto final a jusante da transcrição e tradução, e é, portanto, mais próximo do fenótipo.

Atualmente, intensificou-se a busca por biomarcadores metabólitos específicos em tecidos e/ou biofluidos corporais, como o uso da ADMA. Esta é naturalmente um produto do metabolismo humano encontrado na circulação; portanto, inclui-se nos metabólitos dos fluidos corporais. Assim, os biomarcadores que podem ser detectados no soro ou plasma sanguíneo ganharam importância em virtude da sua eficiência em diagnosticar patologias. Contudo, apesar dos avanços, para Zhao et al.^73^, ainda existem limitações tecnológicas, incluindo a falta de um método único para a extensa análise de todo o metaboloma, bibliotecas e bases de dados espectrais limitados, e algumas desvantagens de *software* para o processamento de dados e extração de biomarcadores. Os autores ainda destacam a necessidade de encontrar um método razoável para análise de metabólitos que possa substituir ou complementar o método tradicional de diagnóstico.

De acordo com Kessler et al.^74^, os recentes avanços na biologia molecular destacam as possibilidades de prevenção e terapia, uma vez que os elevados rendimentos e as estatísticas de genotipagem permitiriam, no futuro, a identificação e intervenção sobre um *loci* genômico, com o potencial de alterar o risco de DCV e, consequentemente, aumentar a prevenção e terapia para os indivíduos. Isso corrobora os achados de O’Donnell e Nabel^75^, que, em estudos de associação do genoma (GWAS), descobriram *loci* genéticos associados com DCVs, incluindo biomarcadores sanguíneos.

Segundo Zhao et al.^76^, a maioria dos testes clínicos usam métodos que incluem testes com um único biomarcador, histopatologia e imuno-histoquímica. Os autores também relatam que os métodos de ensaio recentes são, geralmente, pouco específicos e sensíveis para uma doença em particular, e os biomarcadores tradicionais só mudam significativamente após a lesão ou disfunção substancial da doença.

O uso clínico de biomarcadores é prejudicado em adultos com doença cardíaca congênita, sendo o diagnóstico e o acompanhamento do tratamento frequentemente baseados em testes de exercício cardiopulmonar^77^. Além disso, são usadas categorias de risco bastante rudimentares, que foram identificadas há mais de 50 anos^78^.

O biomarcador ideal, no geral, precisa apresentar certas características (confiável, sensível e específico para a doença), mostrar variação mínima e expressar um baixo nível de base nos indivíduos saudáveis e um alto nível na presença das doenças. Além disso, um biomarcador útil deve ser quantificado por métodos simples e relativamente baratos com resultados reprodutíveis em vários laboratórios^79^.

## CONSIDERAÇÕES FINAIS

Deve-se ressaltar que as DCVs possuem uma complexa etiologia, envolvendo vários fatores, interações e interrelações para promover os mecanismos fisiológicos. Os esforços para o desenvolvimento do diagnóstico se concentram na identificação de biomarcadores, já que há uma crescente necessidade de criar métodos não invasivos para avaliar, monitorar e ajustar a função endotelial e a hipertensão.

Neste artigo, evidenciou-se um conjunto de interações entre a regulação, o metabolismo e a determinação dos níveis intracelulares de ADMA e da enzima NOS ao desempenharem um papel patogênico nas DCVs. Também foi observado o papel de outros potenciais marcadores, como a CPE, a troponina T, a vitamina D e o AU. Precisa ser destacado que alguns estudos indicam os biomarcadores como uma predição de eventos cardíacos, enquanto outros reportam que eles contribuem relativamente pouco para a avaliação, especialmente em populações de baixo risco. Outra consideração é quanto à especificidade dos biomarcadores: a seleção e combinação desses é essencial devido à correlação entre os mecanismos de ação, que desperta o interesse da comunidade científica devido à redução do risco cardiovascular, podendo ser uma alternativa para validar o teste devido à pouca especificidade para o diagnóstico da hipertensão.
