# Supplementary material for: Biomarcadores de função endotelial em doenças cardiovasculares: hipertensão
Source: J Vasc Bras. 2016 Jul-Sep;15(3):224–33. [Article in Portuguese] doi: 10.1590/1677-5449.000316 (PMC5829760; doi:10.1590/1677-5449.000316)
Supplement: Translation

# Translation: Biomarkers of endothelial function in cardiovascular diseases: hypertension

**Authors:** Josynaria Araújo Neves, Josyanne Araújo Neves, Rita de Cássia Meneses Oliveira

**Affiliations:** 1 Universidade Federal do Piauí – UFPI, Núcleo de Pesquisa em Plantas Medicinais – NPPM, Teresina, PI, Brazil.

**Keywords:** biomarkers, hypertension, endothelial function, ADMA

## Abstract

The incidence of systemic arterial hypertension is increasing worldwide. The foundation of prevention is identification of people with hypertension. Nowadays, biomarkers are used to diagnose and stratify diseases and estimates prognosis. The objective of this study was to review articles published over the last 5 years on the subject of biomarkers of cardiovascular diseases. The PubMed, SciELO, Science Direct and MEDLINE databases were searched using the keywords: arterial hypertension, cardiovascular biomarkers, nitric oxide, endothelial function and asymmetric dimethylarginine. The studies reviewed show that cardiovascular diseases have complex etiologies. This article describes evidence demonstrating interactions between nitric oxide and asymmetric dimethylarginine that are involved in regulation, in metabolism, and in determination of intracellular levels, and also discusses other biomarkers related to hypertension. Some studies indicate that biomarkers are useful tools for prediction of cardiac events, whereas others state that they have little to contribute to assessments. Careful selection of tests and combinations of tests may be the key to validating use of biomarkers, in view of their low specificity for diagnosing hypertension.

## INTRODUCTION

In view of its dimensions, the risks involved, and the difficulty of controlling it, systemic arterial hypertension (SAH) can be considered a severe public health problem and one that is associated with a high mortality rate, since it predisposes patients to the development of cardiovascular diseases (CVDs).^1^ Cardiovascular diseases affect more than 83.6 million North Americans and, in Brazil, the Ministry of health registered 326,000 deaths caused by these diseases during 2010, corresponding to around 1000 deaths per day.^2^

According to Georgiopoulou et al.,^3^ the incidence of SAH has increased worldwide. Durande and Gutterman^4^ state that endothelial cells perform a wide range of homeostatic functions. It has been suggested that the combination of vascular endothelial dysfunction and SAH is related to local and systemic inflammation.^5^ It is known that inflammation is a physiological response to protect against harmful and/or pathogenic stimuli and that endothelial dysfunction is a proinflammatory state involving changes to endothelial functions and that it is associated with SAH, which in turn is a multifaceted disease.

Free radicals are among the pathological factors involved, provoking tissue damage and endothelial dysfunction by disturbing the nitric oxide (NO) equilibrium, causing elevated oxidative stress, high proinflammatory cytokine levels (tumor necrosis factor alpha - TNF-α; interleukins IL-6 and IL-1β) and excessive production of inflammatory chemokines [macrophage inflammatory protein alpha-1 (MIP-1α) and monocyte chemoattractant protein-1 (MCP-1)].^6^^,^ 7

Systemic arterial hypertension is a pathology that constitutes a risk factor that is associated with high rates of morbidity and mortality because it contributes to exacerbation of other CVDs and kidney diseases.^8^ As such, SAH is clearly associated with development of vascular lesions and the emergence of dysfunctions in target organs such as the brain, heart, blood vessels, and kidneys.^9^

Biomarkers are widely employed in clinical cardiovascular medicine both for diagnosis and stratification of risk and also for estimating the prognosis of these pathologies.^10^ One example of an application of biomarkers is testing osteoprotegerin levels in cases of heart failure, which may be related to SAH as a cause of hypertrophy of the myocardium.^11^

The objective of this study is to conduct a review of articles in the scientific literature related to the subject of SAH and biomarkers. Over the last 10 years, asymmetric dimethylarginine (ADMA) has emerged as a promising cardiovascular biomarker. Therefore, searches were run for reports relating to plasma ADMA as a new biomarker of putative cardiovascular risk, such as in hypertension, and also for reports describing the contributions made by incorporation of other models of biomarkers, such as endothelial stem cells, troponin T, vitamin D, and uric acid.

A review of the current literature was conducted using bibliographic references published between 2010 and 2015, identified by searching the PubMed, SciELO, Science Direct, and MEDLINE databases, using the following keywords and combinations of them: arterial hypertension, cardiovascular biomarkers, nitric oxide, endothelial function, and asymmetric dimethylarginine. Searches were run for these terms in both Portuguese and in English.

## ARTERIAL BLOOD PRESSURE AND HYPERTENSION

Arterial blood pressure (BP) can be defined as the force exerted by the blood against a given unit of area of the vascular wall. This pressure is generated by the heart and it is the force (potential energy) that enables blood flow and tissue perfusion. Blood flow through the circulation provides organs and tissues with oxygen according to need and removes the metabolites produced by cell activity. Maintenance of an appropriate blood pressure is therefore of fundamental importance for the circulatory system to function well.

Understanding the pathophysiology of SAH requires an understanding of the mechanisms that control BP, which is regulated by activities that are integrated between the cardiovascular, renal, neural, and endocrine systems, and by physical factors such as the force of the heart’s contractions and the elasticity of the major thoracic arteries. According to Franceschini et al.,^8^ maintenance of BP within the normal range is dependent on variations in cardiac output and peripheral resistance, with countless substances and physiological systems interacting in a complex manner to guarantee adequate BP levels in a diverse range of scenarios.

Systemic arterial hypertension is a circulatory disorder.^12^ This multifactorial clinical condition is associated with metabolic and functional/structural abnormalities of target organs (heart, kidneys, and blood vessels), characterized by sustained elevated BP levels, generally above the target levels (systolic pressure ≥ 140 mmHg and/or diastolic pressure ≥ 90 mmHg).^13^^,^ 14 Systemic arterial hypertension, or elevated BP, is recognized as an important risk factor for cardiovascular morbidity and mortality,^15^^,^ 16 even when patients are taking antihypertensive medications.

The literature lists certain environmental factors that have been linked with progression and complications of cardiovascular pathologies, including smoking, obesity, inactivity, and others. The combinations of these factors in people with hypertension appear to vary with age, physical inactivity, hyperglycemia, and dyslipidemia, while obesity increases the prevalence of associations with multiple risk factors.^14^ Lima et al.^17^ conducted a study of Brazilian myocardial revascularization patients and concluded that arterial hypertension, obesity, and inactivity were the most common factors and that 79.5% of the patients exhibited at least three risk factors. It has also been observed that the factors that provoke changes to BP are emerging in ever-younger age groups.^18^

Nobre et al.^19^ point out that the genesis of hypertension encompasses genetic, environmental, vascular, and neural elements. Hyperactivity of the sympathetic nervous system has been proposed as an important mechanism of hypertension and CVD.^20^

Social determinants, such as urbanization, income, aging, and education, influence development of the pathology.^12^ As a result, national and international guidelines for prevention of SAH recommend lifestyle changes, and this approach has gained support over recent years as survival of patients with chronic diseases has increased.^15^

The vascular endothelium plays a role in the pathophysiologic mechanisms leading to SAH.

## ENDOTHELIUM AND PATHOPHYSIOLOGIC CHANGES

In physiological conditions, endothelial cells are controlled by hemodynamic factors such as BP and blood flow, leading to responses that are dependent on production of chemical mediators and that result in changes to blood flow.^21^ These cells play a fundamental role in regulation of vascular tone by synthesis and release of relaxation and contraction factors involved in cardiovascular homeostasis.^22^

According to Days et al.,^23^ the most important endothelium-derived relaxation factors are NO, endothelium-derived hyperpolarizing factor (EDHF), and prostacyclin (PGI2). The principal contractile factors are prostaglandin H2 (PGH2), thromboxane A2, angiotensin II (Ang II), reactive oxygen species (ROS), and endothelin (ET-1).

Endothelium is an organ with multiple functions. Endothelium’s ability to modulate the vascular lumen by regulating vascular tone (controlling local dilation and contraction) or by releasing vasoactive factors is one of the primary responses to physiological stimuli generated by blood flow and blood pressure.^24^^-^ 27

In hypertension, the complex mechanism that raises BP via NO deficiency involves increase of sympathetic system tone, of the renin-angiotensin system (RAS), and of oxidative stress.^28^^,^ 29 All cells, including endothelial cells, have complex enzymatic and non-enzymatic antioxidants systems that act in synergy to defend the organism from damage caused by free radicals.^30^

Nitric oxide is an inorganic, gaseous, highly reactive free radical that is produced by oxidation of the amino acid L-arginine, which is converted into L-citrulline.^23^ Nitric oxide is generated from L-arginine by endothelial NO-synthase in the presence of cofactors. Once produced, it diffuses to vascular smooth muscle cells and activates guanylate cyclase (GC), which results in vasodilation mediated by release of the second messenger cyclic guanosine monophosphate (cGMP) and activation of G-dependent protein kinase, resulting in a reduction in intracellular calcium concentration, followed by vasorelaxation. In addition to this activity, NO has many other functions that take part in regulation of transcription of genes, translation of mRNA, and protein modification of several enzymes involved in mitochondrial respiration, in mitogenesis, and in growth.^31^ The NO and cGMP signaling system is a well-characterized modulator of cardiovascular function in general and of BP in particular^32^ (Figure 1).

**Figure 1:** Endothelial signaling and mechanism of relaxation or contraction of smooth muscle. eNOS: endothelial nitric oxide synthase enzyme; NO: nitric oxide; GC: guanylate cyclase; cGMP: cyclic guanosine monophosphate; Ca^2+^: calcium ions; ECE: endothelin conversion enzyme; ET-1: endothelin 1; ETA, ETB: receptors for endothelins – ETA and ETB in vascular smooth muscle cells; O_2_: free radicals; ROS: reactive oxygen species; ADMA: asymmetric dimethylarginine; DDAH: dimethylarginine dimethylaminohydrolase.

There are three isoforms of the enzyme responsible for nitric oxide synthesis (NOS). The neuronal form (nNOS), found in neurons and activated by calcium, the inducible form (iNOS), stimulated by inflammatory cytokines, microbial products, and mechanical disturbance,^33^ and the enzyme endothelial nitric oxide synthase (eNOS), located on chromosome 7 (7q35-36) are responsible for synthesis of NO in the circulation, with vasoprotective and vasodilatory capacities.^34^ In addition to inhibiting proliferation of smooth muscle cells, they prevent recruitment, adhesion, and differentiation of inflammatory cells, platelet aggregation, and production of thrombogenic thromboplastin.^21^^,^ 35

The activity of eNOS and nNOS is dependent on the calcium/calmodulin complex (Ca^2+/^CaM). As such, it is controlled by variations in the concentration of intracellular calcium, which is an important cytoplasmic signaler. In contrast, iNOS is independent of increases in intracellular Ca^2+^ concentrations, according to Forsterman and Sessa,^31^ and it is expressed in abnormal cellular processes induced and/or stimulated by cytokines, inflammatory agents, and mechanical disturbance, resulting in elevated NO flow.^23^^,^ 34

## BIOMARKERS OF NO/ADMA

Defects of endothelial function and of NO production have been linked with atherosclerosis, hypertension, diabetes, obesity, inactivity, smoking, advanced age, low bioavailability of L-arginine, and presence of infectious agents.^36^

Studies with metabolites of NO, such as nitrite/nitrate (NOx), show that they act as monitors of the state of health of patients with CVDs and can be used as biomarkers in clinical settings.^37^ Rajendran et al.^38^ provided further support for this approach, assessing endothelial function in terms of circulating endothelial biomarkers measured in plasma because there are many attractive candidates for endothelial biomarkers. However, they also pointed out that for many pathologies these molecules offer weak selectivity and specificity and so if they are used individually they have little predictive value.

Considering that production of NO has been associated with low bioavailability of L-arginine,^36^ and that this substrate is an analog of ADMA, studies have suggested that the L-arginine/ADMA ratio has a direct impact on bioavailability of NO.^39^ According to Sharma et al.,^40^ the L-arginine/ADMA ratio in plasma from healthy people is approximately 100:1. However, in a pathophysiologic situation, ADMA exhibits higher concentrations when compared to L-arginine and so ADMA may act to competitively inhibit eNOS, since it prevents formation of NO and, consequently, reduces synthesis of this substrate.^41^ Elevated ADMA levels may therefore inhibit NO synthesis, compromising endothelial function.

High plasma ADMA levels have been reported in patients with vascular risk factors and have a high predictive value for cardiovascular and cerebrovascular events, while Nishiyama et al.^42^ identified ADMA as a biomarker of arterial hypertension. The results of this bibliographic review lead to the conclusion that there have not been enough studies of the effects of intracellular ADMA levels on hypertension in the last 5 years.

Studies have also revealed that elevated/high ADMA levels reduce the participation of NO in regulation of vascular tone by acting to directly inhibit eNOS and to reduce its bioavailability by increasing the production and release of ROS by activation of the RAS, leading to vascular dysfunction in isolated arterial vessels in vitro.^41^

There is evidence to suggest that the imbalance in NO and ROS levels (reduced and increased, respectively) activates the sympathetic nervous system, which is a mechanism that appears to be involved in the neurogenic aspects of hypertension.^43^ Therefore, ADMA could be used as a biomarker, indicating a reduction in the bioavailability of NO.^41^

## BIOSYNTHESIS OF ADMA

The substrate ADMA is formed after proteolysis of proteins containing methylated residues of arginine. This methylation is facilitated by the enzyme methyltransferase (PRMTs), which uses the S-adenosylmethionine (SAM) protein as the methyl donor group.^44^ It is known that there are two types of PRMTs, differentiated according to their specific catalytic activity: type 1 catalyzes formation of ADMA and NG-monomethyl-L-arginine (L-NMMA); while type 2 catalyzes formation of symmetrical dimethylarginine (SDMA) and L-NMMA (Figure 2).

**Figure 2:** ADMA pathway and its relationship with NO. ADMA: asymmetric dimethylarginine; SAM: S-adenosylmethionine; SAH: S-adenosylhomocysteine hydrolase; PRMT1: enzyme methyltransferase type 1; SDMA: symmetrical dimethylarginine; L-NMMA: NG-monomethyl-L-arginine; RAS: renin-angiotensin system; ROS: reactive oxygen species; eNOS: endothelial nitric oxide synthase enzyme; NO: nitric oxide synthesis.

Currently, there is interest in degradation of ADMA by dimethylarginine dimethylaminohydrolase (DDAH),^45^ which is constituted by two isoforms (DDAH-1 and DDAH-2). Degradation of ADMA by the enzymatic isoform DDAH-1 takes place in tissues^46^ and it has been suggested that modulation of DDAH-1 activity by agonists of the farnesoid X receptor (FXR), for example -(2,6-dichlorophenyl)-4-(3'-carboxy-2-chlorostilben-4-yl) oxymethyl-5-isopropylisoxazole (GW4064), could be used as a therapeutic target in treatments for congestive heart failure and other CVDs.^47^

According to Caplin et al.,^48^ 80 to 90% of ADMA is primarily metabolized by DDAHs. There is an alternative route via alanine-aminotransferase 2 glyoxylate (AGXT2) in the kidneys.^49^ Since ADMA accumulates in plasma, it could be considered a pathophysiologic cofactor in cardiovascular and kidney diseases.

According to Davids et al.,^50^ it should be borne in mind that in some cases the levels of ADMA in circulation do reflect intracellular concentrations. However these levels are not in equilibrium.^51^

According to Nemeth et al.,^45^ elevated ADMA levels in the pericardial liquid of cardiac patients may be indicative of important pathophysiologic mechanisms, such as reduction in bioavailability of NO, contributing to the development of cardiac hypertrophy and remodeling. These authors therefore propose that analysis of this liquid could be used as a diagnostic tool because of interference in the content and effects of pericardial liquid, opening up new treatment options to beneficially modify cardiac function and structure.

It is known that ADMA has been identified as a risk factor of endothelial dysfunction acting in several different cardiovascular diseases, accelerating their progression.^52^ Recent studies in patients with chronic kidney disease (CKD) suggest that there is a relationship or association between ADMA levels and fibroblast growth factor 23 (FGF-23), and also a relationship with markers of endothelial cell damage.^53^^,^ 54

Several different studies have assessed conventional and innovative biomarkers for prediction of cardiovascular events. Reriani et al.^55^ point out that there is a need to evaluate the utility of putative biomarkers for evaluation of cardiovascular risk, when compared with endothelial function, because of the small additional value offered by these biomarkers when compared with conventional risk factors. There is speculation that the biomarkers in these studies may only have a minimal role to play in stratification of cardiovascular risk.

## OTHER BIOMARKERS

Table 1 shows a list of some well-known biomarkers. According to Hirata et al.,^56^ many different markers have been cited in the literature on vascular endothelial dysfunction, including insulin, adiponectin, vasodilators (nitrite and nitrate), and vasoconstrictors (ROS, endothelin, thromboxane A2). Some of the studies reviewed for this article were focused on endothelial function and investigation of other possible biomarkers. These are described in the following subsections.

**Table 1:** Some of the markers assayed exhibited relationships with cardiac risk.

Category	Biomarker	Effects
Vascular inflammation	CRP^*^ (protein)	- Provokes endothelial dysfunction by direct suppression of NO production^†^ - Activation of the RAS^‡^ - Increased CRP levels have been associated with increased risk of hypertension
	IL-6^§^, IL-1||, TNF-α^¶^, MCP-1^**^ (cytokine)	- IL-6 has a positive correlation with systolic and diastolic pressures - Concentrations of IL-6 and CRP coincide with increased BMI^††^, elevated lipids and other risk factors related to atherosclerosis
Myocardial stress	BNP^‡‡^ and NT-proBNP^§§^ (neuro-hormone)	- Ventricular dilatation and pressure overload on the myocardial wall - Increased glomerular filtration rate - Increased excretion of water and sodium
Oxidative stress and renal function	Homocysteine	- Increased plasma homocysteine as a marker of cardiovascular risk associated with age, cholesterol and hypertension
Fibrinolytic potential	PAI-1||||	- Inhibitor of serine protease produced by endothelial cells - Increased levels of PAI-1 in hypertensive people caused by induction of shear stress and/or endothelial activation

* CRP: C-reactive protein.

† NO: nitric oxide.

‡ RAS: renin-angiotensin system.

§ IL-6: interleukin-6.

|| IL-1: interleukin-1.

¶ TNF-α: tumor necrosis factor-α.

** MCP-1: monocyte chemoattractant protein-1.

†† BMI: body mass index.

‡‡ BNP: natriuretic peptide type B.

§§ NT-proBNP: pro-BNP.

|||| PAI-1: plasminogen activator inhibitor 1.

### Endothelial Stem Cells (ESCs)

Endothelial stem cells are mononuclear cells that express a combination of endothelial markers (VEGFR2) and progenitors (CD34/CD133) and have a high proliferative capacity. They can be isolated from peripheral blood, bone marrow, and umbilical cord blood. There is no single specific marker that can be used to identify the cells and currently the most widely accepted method is coexpression of the markers CD133, CD34 and VEGFR2. While these markers are not exclusive to ESCs, their presence in combination is characteristic of a specific type of progenitor cell at a specific stage of maturity.^57^^,^ 58

It has been found that ESCs help in endothelial repair processes and the formation of new vessels, which makes them candidates to be used as markers of cardiovascular health.

### Troponin T (hs-cTnT)

McEvoy et al.^59^ conducted an assessment of the sensitivity of troponin T (hs-cTnT), which is a marker of subclinical myocardial injury that could possibly identify individuals at risk of hypertension or left ventricular hypertrophy. They concluded that hs-cTnT was associated with the incidence of hypertension and with risk of left ventricular hypertrophy in a clinical population with no history of CVD. However, they emphasized the need for further research to determine whether hs-cTnT could identify, and benefit clinical screening of, BP or hypertension.

Authors such as Bugnicourt et al.^60^ and Tu et al.^61^ have demonstrated that Troponin concentrations could predict a new onset of atrial fibrillation, since they are independently associated with worse clinical outcomes due to severe adverse cardiac events. The exact mechanism by which atrial fibrillation leads to elevated hs-TN is not fully understood and further studies are needed. However, biomarkers such as hs-TNI or hs-TNT are easy to handle and impose lower financial costs.

Use of hs-TN could identify patients at very high risk and, consequently, allow them to be allocated special treatment with a greater degree of control and definition of stricter limits of possible risk factors, such as BP, high cholesterol, and diabetes mellitus.^62^ It is known that hs-TNI levels are very sensitive for identification of damage to tissues in the heart.

### Vitamin D

The effects of vitamin D on calcium homeostasis and bone metabolism have received a great deal of emphasis in the literature. However, more recent studies have reported that it is associated with several different health problems, including effects on cardiovascular health, since vitamin D can interact with many different mechanisms.

According to the authors Brondum-Jacobsen et al.,^63^ Grandi et al.,^64^ Sokol et al.,^65^ and Wang et al.,^66^ vitamin D levels can be used as biomarkers in patients with CVDs.

The principal vitamin D in circulation is in the form of 25-hydroxyvitamin D. This circulating form binds directly to the vitamin D receptor (VDR) to exert its effect, or it can be converted in the kidneys by 1 α-hydroxylase to 1.25-dihydroxyvitamin D, also known as the hormone calcitriol.^67^^,^ 68 It should be remembered that calcitriol acts by binding with VDR, which is found in the vascular endothelium, vascular smooth muscle, and the myocardium, which raises the possibility of a direct biological effect of vitamin D in the cardiovascular system. The majority of studies report that low levels of 25-hydroxyvitamin D are associated with cardiovascular risk factors such as hypertension, diabetes, and inflammation.

### Uric Acid (UA)

Uric acid is currently one of the areas of greatest interest in research related to SAH. Yanik and Feig^69^ have demonstrated an association between hyperuricemia and arterial hypertension, providing a basis for using serum levels as a biomarker for diagnosis.

Uric acid is formed by breakdown of adenosine and guanine via hypoxanthine, which is converted into xanthine and UA by xanthine oxidase.^70^ The by-products of reactions catalyzed by xanthine oxidase include ROS such as the superoxide anion, which reacts with protons and NO to generate new ROS. These, in turn, provoke damage to the cardiovascular endothelium and microvasculature.^71^

Mamas et al.^72^ point out that the majority of biomarkers are not metabolites, like the troponins, which are proteins. Analysis of the metabolites in bodily fluids has become an important part of diagnosis and of estimation of prognosis and assessment of therapeutic interventions in clinical applications, since the metabolome is the final product, downstream of transcription and translation, which means that is more closely related to phenotype.

Currently, the search for biomarkers in the form of specific metabolites in tissues and/or bodily fluids is very intense. One such, ADMA, is naturally a product of human metabolism that is found in circulation and is therefore one of the metabolites found in bodily fluids. Biomarkers that can be detected in blood serum or plasma have gained importance because of their efficacy for diagnosis of pathologies. However, despite all of the advances that have been made, Zhao et al.^73^ point out that there are still technological limitations, including the lack of a single method for extensive analysis of the entire metabolome, limited spectral libraries and databases, and certain disadvantages of the software available for processing data and extracting biomarkers. The same authors also point to the need to find a reasonable method for analysis of metabolites that could substitute or complement the traditional method of diagnosis.

According to Kessler et al.,^74^ recent advances in molecular biology have highlighted the possibilities for prevention and treatment, since the high yields and the statistics of genotyping will, in the future, enable identification of, and intervention in, specific genomic loci with the potential to change the risk of CVD and, consequently, increase prevention and treatment options for individual people. This is in line with findings published by O’Donnell and Nabel,^75^ who conducted genome-wide association studies (GWAS), discovering genetic loci associated with CVDs, including biomarkers in the blood.

According to Zhao et al.,^76^ the majority of clinical tests use methods that include tests with a single biomarker, histopathology, and immunohistochemistry. The same authors also state that recent assay methods are generally of low specificity and sensitivity for a given disease in particular and that the traditional biomarkers only change significantly once the disease has caused substantial damage or dysfunction.

Clinical use of biomarkers is compromised in adults with congenital cardiac disease and diagnosis and follow-up of treatment are commonly based on tests of cardiopulmonary exercise.^77^ Additionally, the risk categories employed are very rudimentary and were identified more than 50 years ago.^78^

In general, the ideal biomarker should offer certain characteristics (reliability, sensitivity, and specificity for the disease), should exhibit minimal variation and should have a low baseline level in healthy people and a high level in the presence of diseases. Additionally, a useful biomarker should be quantifiable using simple and relatively inexpensive methods with results that are reproducible in many different laboratories.^79^

## FINAL COMMENTS

It should be stressed that CVDs have complex etiology, involving many different factors, interactions, and interrelations, that in combination provoke the physiological mechanisms. Efforts to develop diagnostic methods are concentrating on identification of biomarkers, since there is a growing need for noninvasive methods to evaluate, monitor, and adjust endothelial function and hypertension.

In this review evidence was compiled showing a large number of interactions between regulation, metabolism, and determination of intracellular levels of ADMA and of the NOS enzyme that play pathogenic roles in CVDs. Evidence was also identified showing that other potential markers, such as ESCs, troponin T, vitamin D, and UA, also play roles in these processes. It should be stressed that while some of the studies reviewed indicate that biomarkers have potential for prediction of cardiac events, others state that they contribute relatively little to assessments, especially in low-risk populations. Another issue identified is related to the specificity of biomarkers: it is essential to select and combine them because of the correlations between mechanisms of action, and this has attracted the interest of that section of the scientific community that is focused on reduction of cardiovascular risk, since it could be a viable option for validating these tests, in view of their low specificity for diagnosis of hypertension.
